# Supplementary material for: The MTL200: A surface-based, probabilistic atlas of the medial temporal lobe
Source: Imaging Neurosci (Camb). 2026 Nov 9;4:IMAG.a.1447. doi: 10.1162/IMAG.a.1447 (PMC13749375; doi:10.1162/IMAG.a.1447)
Supplement: Supplementary file 1 [file IMAG.a.1447_supp.pdf]

Supplementary Information

Supplementary Information for Faul et al. “The MTL200: a surface-based, probabilistic atlas of the medial temporal lobe.”

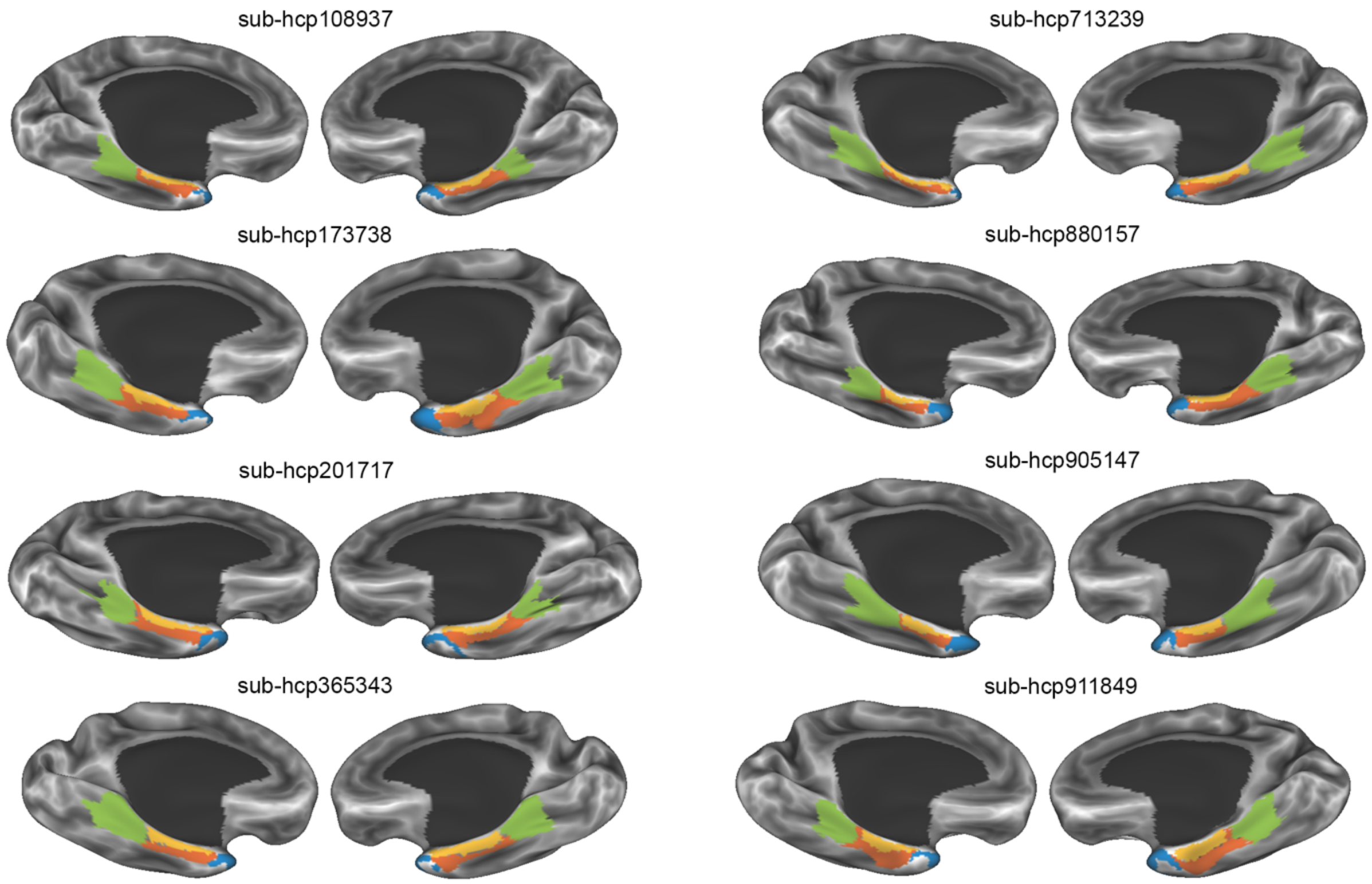

Fig S1. Parcellations on the cortical surface in eight example participants (medial view).

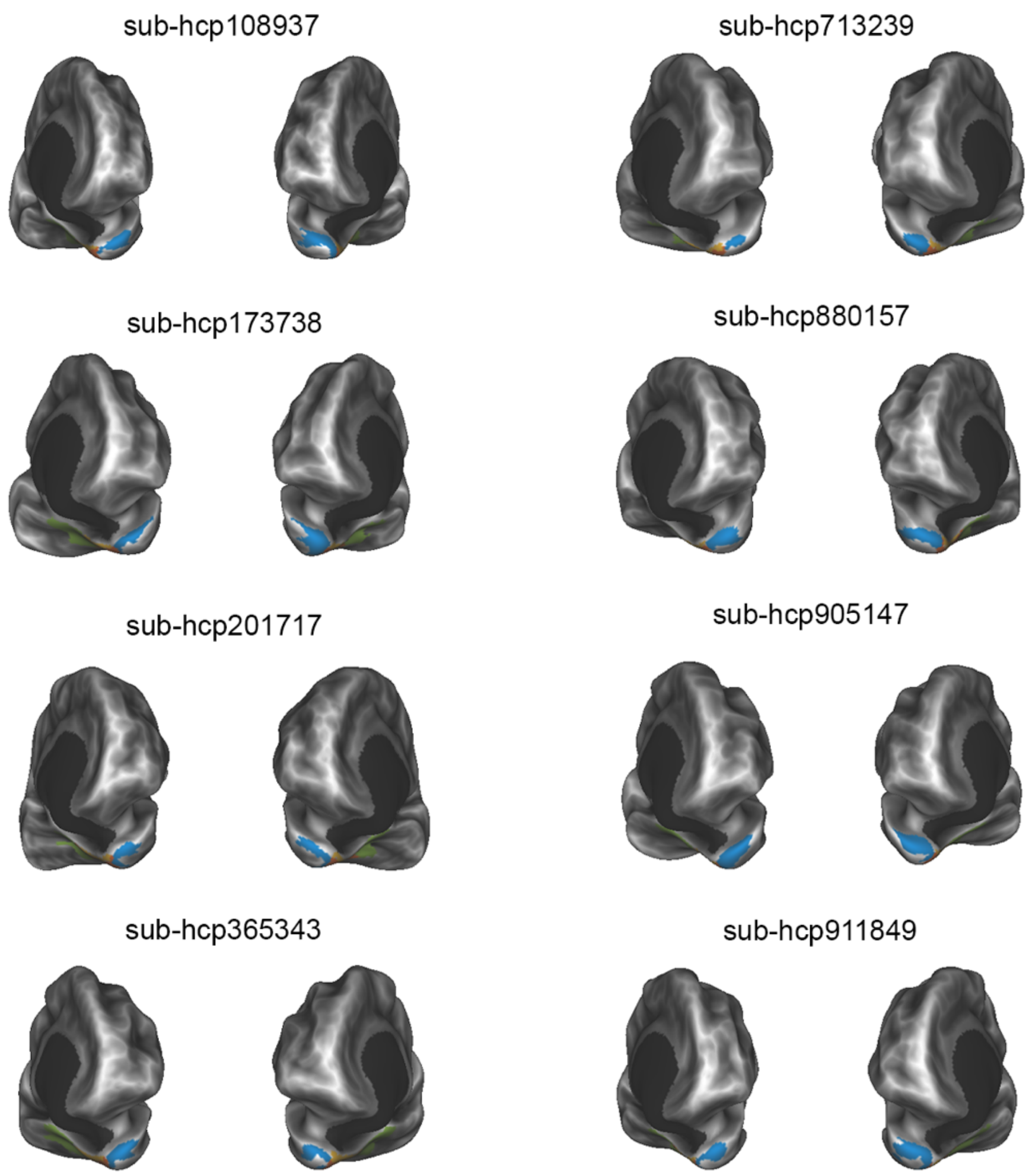

Fig S2. Parcellations on the cortical surface in eight example participants (frontal view).

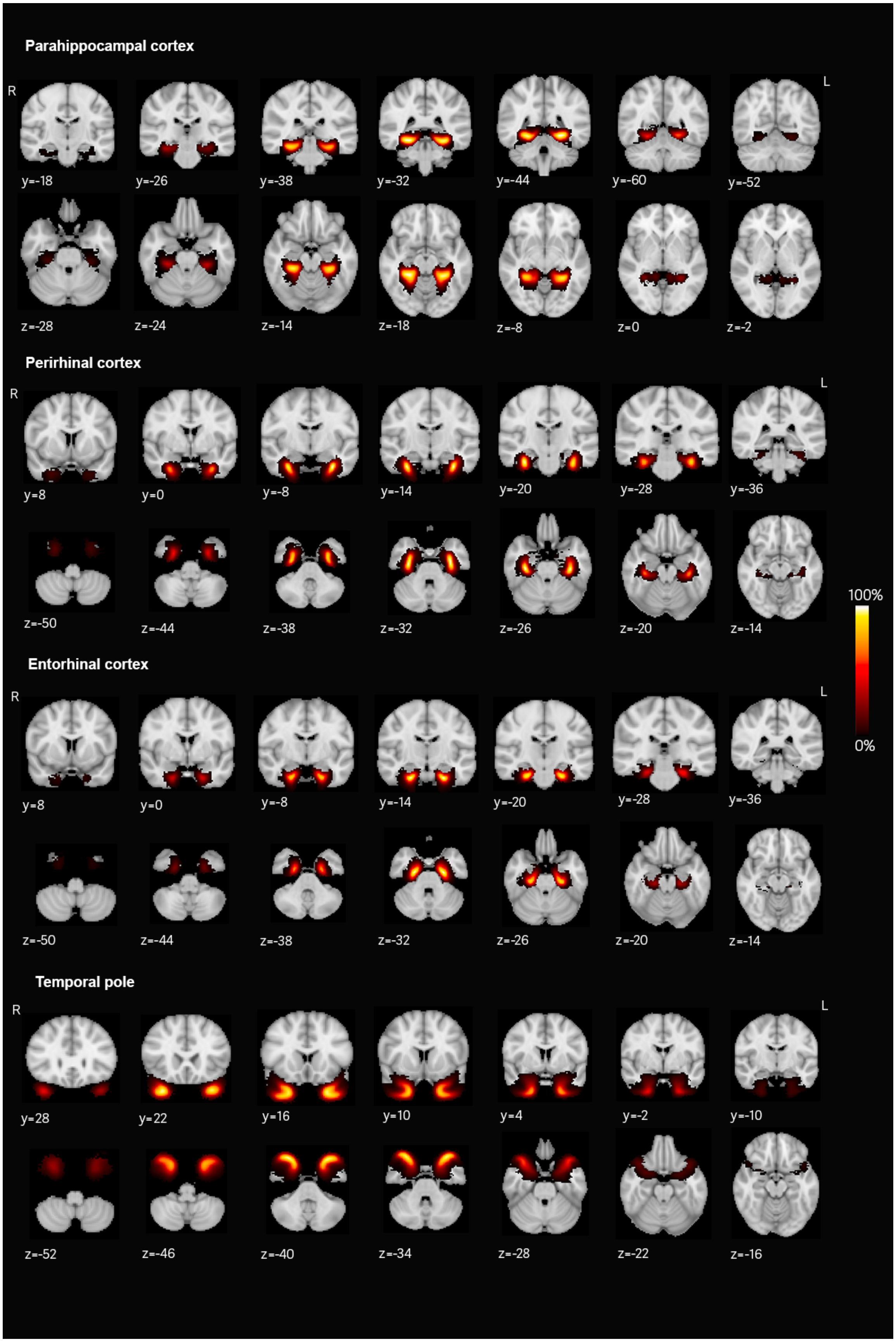

Parahippocampal cortex
R
L
y=-18
y=-26
y=-38
y=-32
y=-44
y=-60
y=-52
z=-28
z=-24
z=-14
z=-18
z=-8
z=0
z=-2
Perirhinal cortex
R
L
y=8
y=0
y=-8
y=-14
y=-20
y=-28
y=-36
z=-50
z=-44
z=-38
z=-32
z=-26
z=-20
z=-14
100%
0%
Entorhinal cortex
R
L
y=8
y=0
y=-8
y=-14
y=-20
y=-28
y=-36
z=-50
z=-44
z=-38
z=-32
z=-26
z=-20
z=-14
Temporal pole
R
L
y=28
y=22
y=16
y=10
y=4
y=-2
y=-10
z=-52
z=-46
z=-40
z=-34
z=-28
z=-22
z=-16

Fig S3. Volumetric probabilistic atlas in MNI152 space.

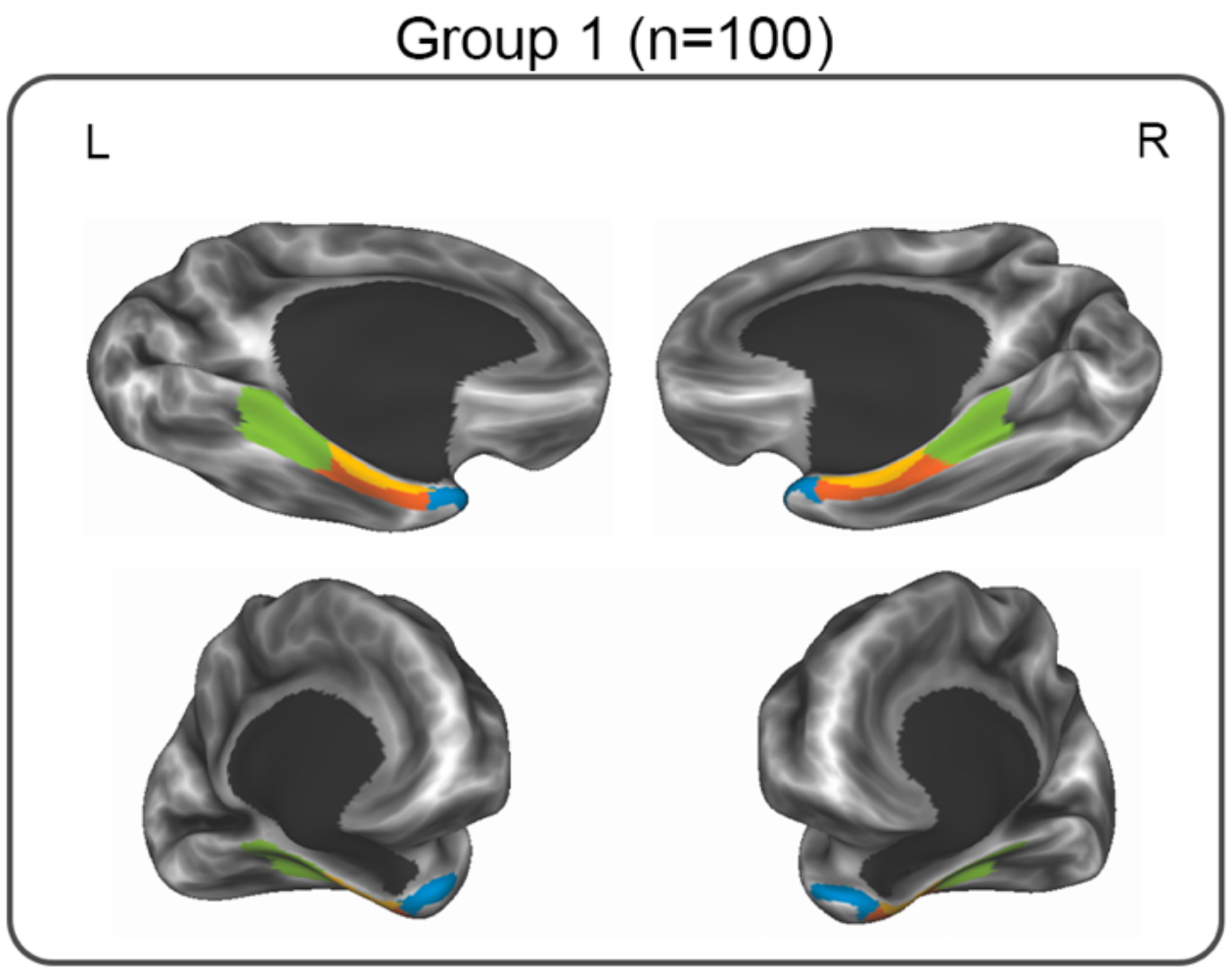

Fig S4. Split-half analysis demonstrates high reliability in the discrete segmentation generated across two partitions of participants.

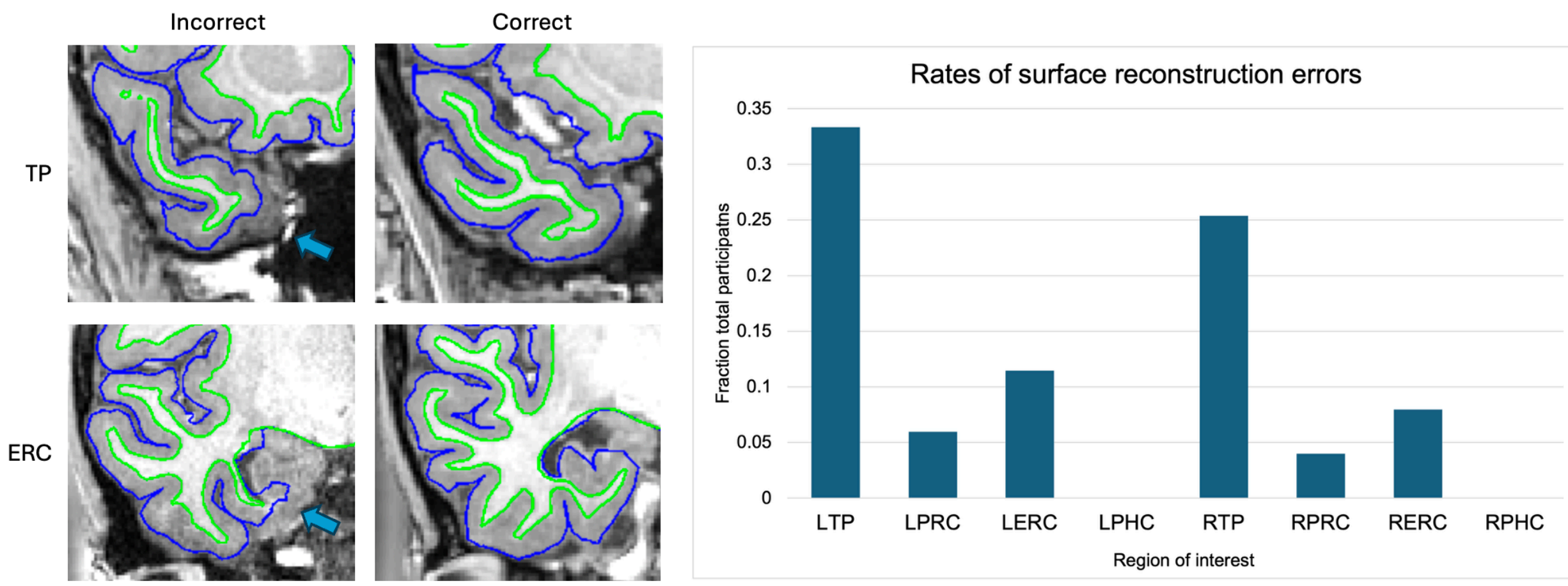

Fig. S5. Left: examples of surface reconstructions with and without reconstruction errors. Right: rates of reconstruction error across participants for each region.
